# Canto: an online tool for community literature curation

**DOI:** 10.1093/bioinformatics/btu103

**Published:** 2014-02-25

**Authors:** Kim M. Rutherford, Midori A. Harris, Antonia Lock, Stephen G. Oliver, Valerie Wood

**Affiliations:** ^1^Cambridge Systems Biology Centre, ^2^Department of Biochemistry, University of Cambridge, Sanger Building, 80 Tennis Court Road, Cambridge CB2 1GA and ^3^Department of Genetics, Evolution and Environment, and UCL Cancer Institute, University College London, Darwin Building, Gower Street, London WC1E 6BT, UK

## Abstract

**Motivation:** Detailed curation of published molecular data is essential for any model organism database. Community curation enables researchers to contribute data from their papers directly to databases, supplementing the activity of professional curators and improving coverage of a growing body of literature. We have developed Canto, a web-based tool that provides an intuitive curation interface for both curators and researchers, to support community curation in the fission yeast database, PomBase. Canto supports curation using OBO ontologies, and can be easily configured for use with any species.

**Availability:** Canto code and documentation are available under an Open Source license from http://curation.pombase.org/. Canto is a component of the Generic Model Organism Database (GMOD) project (http://www.gmod.org/).

**Contact:** helpdesk@pombase.org

## 1 INTRODUCTION

The major activity of any model organism database (MOD) is the manual curation of gene-specific information from peer-reviewed research articles, a time- and labour-intensive process that involves reading publications and associating novel biological information with genes or other biological features. Several factors now motivate databases to develop alternative curation strategies to supplement the efforts of professional curators to maintain comprehensive annotation. Most pressingly, continuing growth in both the number of papers published, and the amount and complexity of information contained in a typical paper, threatens to outstrip the capacity of database staff. In addition, curators’ biological knowledge tends towards breadth rather than depth; a curator may annotate a paper on an unfamiliar topic in less than optimal detail, or make errors that experts would avoid.

PomBase (Wood *et al.*, 2012), the MOD for the fission yeast *Schizosaccharomyces pombe*, has introduced a community curation initiative that engages researchers in direct curation of their publications, addressing issues of both literature volume and specialized knowledge simultaneously. To support this, we have developed Canto, a web-based tool that enables professional curators and publication authors to capture detailed biological knowledge accurately and consistently, using ontologies from the OBO Foundry collection (Smith *et al.*, 2007). Canto can be configured to use gene (or gene product) identifiers for any species, as well as any of several ontologies, and can therefore be readily adapted for diverse uses.

## 2 CURATION INTERFACE

Canto provides a simple, intuitive annotation interface that requires no specialized training for use. The user is guided step-by-step through the annotation procedure, ensuring that all essential, and any optional, data required by a particular MOD are collected.

In Canto, annotation is organized at the level of an individual publication. For any paper, the first curation step is to specify the genes (or gene products) to be annotated. For each gene, the user then selects a type of data to curate. The types of identifiers allowed and the available data types are determined by configuration (see Section 4). Subsequent annotation steps are specific to the data type.

User documentation is provided as web pages and mouse-over tooltips. A destination for user requests, such as a helpdesk address, can also be configured.

### 2.1 Curation using ontology terms

Most curation types in Canto use terms from bio-ontologies. Current Canto implementations use the Gene Ontology (GO) (The Gene Ontology Consortium, 2013) for function, process and component annotations and PSI-MOD for protein modifications (Montecchi-Palazzi *et al.*, 2008). The PomBase Canto instance uses the Fission Yeast Phenotype Ontology (Harris *et al.*, 2013) for phenotype annotation, but any other ontology of precomposed phenotypes can be substituted. To simplify ontology navigation for novice users, details of complex ontology structure are hidden. Instead, the user types familiar search strings, and then selects a relevant general term from a list of matching term names and synonyms. The user is then directed to the most specific applicable child term. Links to external ontology browsers (such as AmiGO and QuickGO) provide access to the ancestry and context of the term.

The interface then guides the user through subsequent steps that gather evidence and additional supporting data. For example, all ontology annotations require evidence, selected from options tailored for the specific ontology. Phenotype annotations capture details of alleles, expression levels and experimental conditions. Finally, annotations can be transferred from one gene to another, streamlining the curation process. Because annotations are made using precise ontology terms without free text input, format and syntax errors are avoided. Users can, however, provide comments pertaining to individual annotations or to the whole article.

### 2.2 Interaction curation

In addition to assigning ontology terms to genes, users can curate genetic and physical interactions. Starting from one gene, the user selects an interaction type (physical or genetic), an interacting gene and an experiment type. Canto is configured to use BioGRID experiment types by default (Chatr-Aryamontri *et al.*, 2013).

### 2.3 Literature and curation management

Canto includes an administrator interface that supports literature- and curation-management tasks. Papers are retrieved from PubMed according to administrator-specified criteria, such as organism or publication date. Administrators can then use the literature triage function to classify papers by type (e.g. curatable, review, methods) and prioritize for curation. Administrators can select and curate papers, or invite authors to curate publications. Users can also select their own papers for curation via a publication search. Administrators can monitor curation progress, amend annotations in any active session and flag curation sessions as approved for public release.

## 3 METHODS

Canto is implemented in Perl using the Catalyst web framework and other widely used Perl packages, and has been engineered to ensure that new annotation types can be added easily. In its standard mode of operation, Canto has no external dependencies, although it can be configured to use web services to retrieve gene and publication details. All data is stored locally using the SQLite library. A CLucene (http://clucene.sourceforge.net/) index of ontology term names and synonyms supplies suggestions to the search autocomplete feature. A small amount of Javascript is used on the browser side to make the application more responsive.

Canto can export in JSON format for loading into databases that use the Chado schema (Mungall *et al.*, 2007), or for archiving or other applications. Curated GO data can be exported in Gene Association File format (Balakrishnan *et al.*, 2013).

## 4 CURRENT IMPLEMENTATIONS

The original implementation of Canto supports community curation for *S.pombe* literature, as part of the PomBase project. Because many aspects of Canto, such as supported ontologies, and gene/gene product identifiers, are fully configurable, Canto can be easily deployed for other organisms, with or without a dedicated organism-specific database. We have set up two additional Canto installations, illustrating its flexibility. In a species-specific example, literature triage for the yeast *Komagataella pastoris* (formerly *Pichia pastoris*) has been completed, and annotation is planned (D. Dikicioglu *et al.*, manuscript in preparation). A species-independent version of Canto supports GO annotation using UniProtKB protein accessions. Current Canto installations, including a demonstration tool, are accessible on the Canto home page (http://curation.pombase.org/).

## 5 FUTURE DEVELOPMENT

Canto will be enhanced to support ontology subsets, taxon restrictions (Deegan *et al.*, 2010) and annotation extensions (R.P. Huntley *et al.*, manuscript in preparation). We will also incorporate semantic checks for logical consistency and comprehensive annotation. To improve efficiency, we will enable Canto to link to TermGenie (http://termgenie.org; H. Dietze *et al.*, manuscript in preparation), which streamlines the creation of new GO terms. To increase interoperability, we plan to provide functionality to export to GPAD (The Gene Ontology Consortium, 2013) and other useful formats as needed.
